# Bronchopulmonary Dysplasia: Ongoing Challenges from Definitions to Clinical Care

**DOI:** 10.3390/jcm12113864

**Published:** 2023-06-05

**Authors:** Sushma Nuthakki, Kaashif Ahmad, Gloria Johnson, Milenka Cuevas Guaman

**Affiliations:** 1 Pediatrix Neonatology of Houston, Houston, TX 77074, USA; 2 Department of Neonatology, The Woman’s Hospital of Texas, Houston, TX 77054, USA; 3 Department of Pediatrics, Division of Neonatology, Texas Children’s Hospital, Baylor College of Medicine, Houston, TX 77030, USA

**Keywords:** bronchopulmonary dysplasia (BPD), chronic lung disease (CLD), neonatal intensive care unit (NICU), gestational age (GA), post-menstrual age (PMA), continuous positive airway pressure (CPAP), high-flow nasal cannula (HFNC), oxygen (O_2_), ventilator

## Abstract

Bronchopulmonary dysplasia (BPD) is the most common complication of extreme prematurity. Its etiology is multifactorial and is attributed to genetic susceptibility to prenatal and postnatal factors. As advancements in neonatology have led to the increased survival of premature infants, a parallel increase in the incidence of BPD has occurred. Over time, the definition and diagnostic criteria for BPD have evolved, as have management strategies. However, challenges continue to exist in the management of these infants, which is not surprising given the complexity of the disease. We summarize the key diagnostic criteria and provide insight into the challenges related to various aspects of BPD definitions, data comparisons, and clinical care implementation.

## 1. Introduction

Bronchopulmonary dysplasia (BPD) is a term used to define chronic lung disease in preterm infants.

Over time, both the definition and pathogenesis have evolved [1,2]; however, gaps continue to exist in standardizing the care of these infants due to the complex nature and spectrum of the disease. Genetic predisposition, prenatal factors, such as maternal hypertension and chorioamnionitis, and postnatal factors, such as barotrauma, volutrauma, and atelectotrauma, are some of the key pathogenic mechanisms described [3,4,5,6,7]. Reaching a consensus on how best to define, diagnose, and measure BPD is critical for several reasons, from classifying the spectrum to answering research questions and individual management and prognostication. The incidence of BPD is also seen as a quality indicator and a reflection of the respiratory care provided in a NICU within an institution or for a network of NICUs, such as Vermont Oxford Network (VON), Neonatal Research Network (NRN), or Children’s Hospital Neonatal Consortium (CHNC). In this article, along with highlighting components of these definitions, we focus on the issues related to the clinical care of these infants.

## 2. Definition of BPD

### 2.1. Brief History/Evolution of Definitions of BPD

BPD is a disease that has essentially been defined by its treatment [8]. The history of the term starts with Northway, who first coined the term in 1967 [9]. The condition then was described as a pathologic process in the lung tissues of infants whose acute respiratory distress syndrome is prolonged, requiring oxygen (O_2_) for hours, along with X-ray and histological findings. Bancalari et al. redefined the diagnosis of BPD as the need for intermittent positive pressure ventilation (IPPV) in the first week of life for more than 3 days and supplemental O_2_ for longer than 28 days [10]. Shennan and colleagues found that regardless of gestational age at birth, the O_2_ requirement at 36 weeks increases the positive predictive value of abnormal outcomes [11].

The refinement of the definition of BPD continued, and in 2001, the NICHD held a consensus conference to generate a comprehensive and standardized definition of BPD that would be widely applicable [12]. The key features of this definition include a continuous O_2_ requirement for the first 28 days and O_2_ requirement at 36 weeks PMA. *The 28-day criterion* definition of BPD requires an infant to receive supplemental O_2_ of >0.21 consecutively for the first 28 postnatal days of life, for at least 12 h each day. This definition also categorized infants into <32 weeks and >32 weeks for minor variations in age-related milestones. The NICHD published recommendations for a revised definition in 2016 to include newer modes of non-invasive ventilation for support and assigned grading from I to III using 36 weeks PMA as a milestone, with Grade III being the most severe form of BPD [13] (Table 1). This definition also included Grade III(A), which was defined as patients whose early death (between 14 days postnatal age up until 36 weeks) was due to parenchymal lung disease and no other neonatal comorbidities.

The BPD Collaborative, which was developed in the last decade, recommended that severe BPD (sBPD) be classified into two subcategories, type 1 sBPD and type 2 sBPD, based on data from Ehrenkranz et al., 2005 [14]. This added value from the management and prognosis perspective to defining the incidence in the world of epidemiology [15].

The analysis of the neonatal research network (NRN) data by Jensen et al. led to the proposal of a definition of BPD according to the level of respiratory support and supplemental O_2_ administered at 36 weeks PMA that best predicted death or serious respiratory morbidity through 18–26 months corrected age and likewise did not use the 28-day criteria. Importantly, this classification eliminated O_2_ as a criterion, and it addressed the predictive value of the 36-week PMA criteria and long-term morbidity and mortality [16].

The next category of definitions based on the phenotypic approach and targeted management has been described by several authors [17,18]. Wu et al. classified BPD into three key subgroups based on pathophysiology, i.e., moderate to severe parenchymal lung disease, vascular component, and airway disease and reported outcomes in each of the categories [19]. Furthermore, this is sub-classified into small airways, central airways and distal space and vasculature along with the suggested management approach [20,21].

### 2.2. Database Definitions

Given the evolving definitions of BPD, as described above, various neonatal databases have extrapolated customized versions of these definitions that take into account individual data extraction and reporting characteristics. The Vermont Oxford Network (VON), Children’s Hospital National Database (CHND), Pediatrix, and BPD Collaborative are some of the largest neonatology databases we are aware of.

The Vermont Oxford Network uses 36 weeks PMA as the milestone for infants less than 33 weeks to report the incidence of chronic lung disease regardless of prior O_2_ [22]. This definition, though it is relatively easier to extract the data, does not necessarily differentiate the spectrum of the disease severity and is less ideal for generalized comparisons across institutions.

In the Pediatrix Clinical Data Warehouse, data can be reported in multiple ways and are utilized by medical and quality improvement leaders based on the information needed. Offered tracking measures include the (a) Jensen grading, (b) the need for supplemental O_2_ at 28 days, (c) the need for supplemental O_2_ at 36 weeks, and (d) clinician reporting of BPD diagnosis.

The BPD Collaborative, which was created in 2012 with seven founding centers, has now grown to 34 centers. It maintains a registry that collects data for infants with severe BPD in both the inpatient and the outpatient settings. The BPD collaborative utilized the NICHD 2001 definition of BPD from 2012 to 2022, at which time they transitioned to the Jensen grading system. Similarly, the CHNC database transitioned to Jensen grading as well.

## 3. Challenges

### 3.1. Challenges with Definition

Comparing BPD rates across publications and databases may overestimate or underestimate the true rate of BPD. The diagnosis of BPD and defining its epidemiology requires accurate, valid, and reliable criteria, along with a consistent application of criteria across centers and databases. Each definition, despite best efforts, has limitations, and as Bancalari stated, the diagnosis of BPD has different meanings for different stakeholders.

The initial comprehensive definition from the NICHD is based on O_2_ requirement and age-related milestones at 28 days and at 36 weeks PMA. Measuring supplemental O_2_ treatment consecutively for the first 28 days requires significant effort. Although some authors have used this definition [14], others have used an extrapolated and pragmatic version [15], i.e., cumulative 28 days or the use of supplemental O_2_ on day 28 of life [23]. Secondarily, infants on nasal CPAP and ventilators were grouped into the same category. The subclassification by Ehrenkanz et al. of sBPD into grade 1 and grade 2 (Table 1) helped differentiate the two groups [14]. However, the practicality of data extraction remains and is expected, given the spectrum and the complicated disease process.

As stated in our previous publication, although Jensen grading did not include the 28-day criterion and has not been thoroughly vetted in validation studies, it is much more refined and straightforward to understand and measure [24]. Additionally, when comparing the prevalence of BPD utilizing the VON definition or the Jensen definition, there is a clear difference (Table 2). Jensen definitions have moved away from FiO_2_ for practical reasons, as the O_2_ requirement of an infant is a number in Electronic Medical Records (EMRs) that can vary on any given day. Some EMRs may have nurses or RTs document a different number of O_2_ requirements throughout the day. Alternatively, the pediatrix CDW only has one FiO_2_ listed per day, and there is no definition as to whether it should be the greatest FiO_2_, lowest FiO_2_, most recent FiO_2_ when the note is written, or something different. Currently, most institutions and databases appear to be transitioning to Jensen grading while waiting for further assessments to determine their usefulness in predicting long-term pulmonary outcomes.

### 3.2. Challenges in Clinical Care

#### 3.2.1. Evolving Patient Population

Given the spectrum of BPD, outcomes are certainly associated with the category of diagnoses, i.e., the more severe the disease, the higher the risk of morbidity. There are reports of increased trends in terms of the survival of preterm infants, including 22,23-weekers and small-for-gestational-age (SGA) infants, along with the incidence of severe BPD [24,25]. The combination of sBPD and SGA infants is a cohort that needs special expertise given the increased risk of comorbidities, including tracheostomies. In places with a lack of interdisciplinary collaboration and expertise within a NICU, it is not uncommon to transfer these patients at a certain gestational age to a pediatric ICU, where caregivers of various disciplines may or may not be familiar with the pathophysiology and who are minimally aware of the management of these infants. Additionally, redirecting the care of some of these infants is not unheard of. Another important point is that the consequences of BPD do not end in the NICU; they continue to evolve in children and adolescents, as well as the risk for other pulmonary diseases in children and/or adults.

#### 3.2.2. Interdisciplinary Team

Given the limited evidence in quite a few aspects of the care of these infants, it is expected to have variation within the practice and across institutions from fluids to ventilator management. Management is multifaceted and requires dedication and passion from all disciplines involved, including physicians and nurse practitioners, nursing, dieticians, respiratory therapists, and pulmonologists. Primary nursing and developmental care of the infant through the growth phase are as critical as the other disciplines. After antenatal corticosteroids and surfactants, the remainder of the evidence is nominal. Henceforth, having a team that can provide a framework of consistent care across all of your BPD patients will improve the quality of care.

#### 3.2.3. Management Strategies

The preventive care of BPD is widely discussed compared to the management aspects of the disease.

The literature taken from various studies and consortiums added to the depth of knowledge, from management based on phenotypes to recent evidence of the potential benefits of superoxide dismutase to protect against oxidant injury and pentoxifylline, a phosphodiesterase inhibitor that suppresses cytokine production [26,27,28,29]. The data produced by Gentyala et al. on the most commonly used medications for BPD management, cumulative days of exposure, and marked variation in outcomes is very compelling [30]. Other authors have also highlighted the variability in the use of diuretics [15,31]. While diuretics reportedly improve short-term lung function and have potential benefits, the judicious use of diuretics, especially in growth-restricted infants, is warranted. A similar pattern is observed with bronchodilator use with marked variation across units, and the BPD collaborative statement recommended that bronchodilator therapy be evaluated clinically and limited to those BPD patients who demonstrate significant positive responses [32].

Evidence for simple and straightforward clinical questions for care providers is much needed to minimize variability and is somewhat lacking. Although the importance of functional residual capacity (FRC) and the relationship with pulmonary vascular resistance (PVR) is widely discussed, interface(s) for nCPAP use and the weaning of nasal CPAP is a topic that is widely disagreed upon. Another popular debate is HFNC vs. low-flow cannula. These are mostly driven based on the preference of the key stakeholders or provider’s preference as the evidence is not available in this population. Other factors described include the lack of standardization of target saturations within units and the influence of co-morbidities [33].

## 4. Discussion

Despite the introduction of the NICHD definition as a standard method of diagnosing and measuring BPD, significant practical challenges and questions persisted in terms of the application of its use for obvious reasons, and as Bancalari stated, diagnosis has different meanings for various key stakeholders [34].

If BPD rates are used to assess the quality of care within an institution or as a performance assessment tool across institutions, a set of definitions based on the goal and necessity is important to make the comparisons valid.

In all current definitions, infants are to be assessed for the presence or severity of BPD at specific time points related to the infant’s age or at discharge. Thirty-six weeks PMA is a commonly used time point in many definitions and databases, as this seems to predict abnormal pulmonary outcomes better than the 28-day criterion. The actual concentration of O_2_ delivered to the infant is known as ‘effective FiO_2_’, a term commonly used in neonatal intensive care units, which varies with minute ventilation, the interface used, and the ratio of mouth-to-nose breathing [2]. It can be calculated from the infant’s weight, the O_2_ L flow, and the O_2_ concentration using published formulae [35,36]. Most clinical documentation in the medical record only describes the delivered FiO_2_ and not the effective FiO_2_. It is a variable that has not been added thus far to the definitions. As technology improves, and if this becomes an automated EMR data point, that may add some value, especially for Jensen classification along with differentiating phenotypes. Several scores, such as Respiratory Severity Score (RSS) and the Pulmonary score, to predict outcomes are reported in the BPD literature as well [37,38,39].

The frequency of the usage of postnatal corticosteroids varies widely across NICUs, from 0 to 87% [15]. Units that have a high rate of usage of postnatal corticosteroids may possibly have a lower rate of BPD. However, the routine use of postnatal corticosteroids to very low birth infants (VLBW) to prevent or treat BPD has been discouraged by the American Academy of Pediatrics and the Canadian Paediatric Society [40,41,42]. One caution while interpreting the incidence of BPD is the concomitant NICU use of postnatal steroids.

Neonatal units should carefully examine their internal practices to eliminate the use of multiple devices that provide positive pressure to minimize variability, e.g., the use of both nCPAP and HFNC can introduce variability depending on the provider’s preference. Over the past decade, collaborative care and efforts have been developed to improve overall outcomes and to minimize and prevent the risk of Cor Pulmonale or pulmonary hypertension.

## 5. Conclusions

We highlight some of the issues and speculate that clinical definitions will continue to evolve as we gain more in-depth knowledge concerning BPD. The authors suggest finding and highlighting clinical solutions in parallel beyond definition to assist care providers in optimizing the management of this vulnerable population. We advocate focusing on identifying tools of success and the barriers in terms of implementation across diverse institutions, from tertiary care to community/satellite hospitals. It is not an easy task to conduct randomized controlled trials (RCTs) to answer every clinical question, given the complexity and rigor of these trials. Collaborating and blending quality initiatives and comparative effectiveness research (CER) studies along with RCTs hopefully will help answer most of the clinical conundrums. Recognizing this subset of infants as a high-risk group by the key stakeholders, prioritizing and allocating resources, and framing guidelines that can be applicable from tertiary care to satellite hospitals are some of the suggested next steps.

## Figures and Tables

**Table 1:** The evolving definitions of BPD.

	Definition	Highlights and Limitations
1967, Northway [9]	Coined the term	
1979, Bancalari [10]	O_2_ requirement at 28 days of life	
1988, Shennan [11]	O_2_ requirement at36 wks PMA	
2001, NICHD Consensus *	O_2_ > 28 DOL and O_2_ requirement at 36 wks PMA **Mild**—RA at 36 wks PMA or at discharge, whichever comes first **Moderate** FiO_2_ < 30% **Severe** FiO_2_ > 30% or PPV or nCPAP	The very first comprehensive definition Difficult to track O_2_ Infants on nCPAP and ventilator were combined
2005, Ehrenkanz [14] BPD Collaborative	**sBPD type 1**, Infants on nCPAP **sBPD type 2**, Infants onVentilator	FiO_2_ not included
2016, NICHD Revised	**Grade I** (nCPAP), (NIPPV), or (HFNC) (≥3 L/min) at 21% O_2_ or NC 1 to <3 L/min, hood O_2_ at 22–29%, or NC < 1 L/min at 22–70% O_2_	Much more detailed
	**Grade II** IPPV invasive positive pressure ventilation (IPPV) at 21% O_2_, nCPAP, nIPPV, or HFNC at 22–29% O_2_, NC 1 to <3 L/min or hood O_2_ at ≥30% O_2_, or NC < 1 L/min at >70% O_2_	Included newer modes of respiratory support
	**Grade III** IPPV > 21% O_2_, and nCPAP, nIPPV, or HFNC at ≥30%	Issue with O_2_ tracking continues
	**Grade IIIa**	Death between 14 days and 36 wks from parenchymal lung disease included
2019, NRN Jensen Grading	**Grade 1** < 2 lpm **Grade 2** nCPAP/NIPPV/>2 lpm **Grade 3** Invasive PPV	Simplified version Does not account O_2_
2020 Phenotype definitions	Parenchymal lung disease Vascular component Airway disease	Based on pathophysiology

PMA: post-menstrual age; wks: weeks; nCPAP: nasal continuous positive airway pressure; NIPPV: nasal intermittent positive pressure ventilation; HFNC: high-flow nasal cannula. * Classified < 32 weeks and >32 weeks. Lpm: litres per minute; FiO_2_: fractionated inspired O_2_; RA: room air; PPV: positive pressure ventilation; IPPV: invasive positive pressure ventilation; NC: nasal cannula.

**Table 2:** Definitions used to report outcomes in various databases.

Database	Definition	Key Points
VON	O_2_ at 36 wks PMA (infants < 33 weeks GA at birth)	Easier to report Severity poorly defined Prevalance 52% *
Pediatrix database	Jensen grading O_2_ at 28 days O_2_ at 36 wks PMA Physician Reporting	Data are reported based on the user requirement Jensen Prevalance 63% *
BPD Collaborative	Jensen Grading	Jensen Prevalance 63% *
CHNC	Jensen Grading	Jensen Prevalance 63% *

VON: Vermont Oxford network; CHNC: Children’s Hospital National Consortium. * From Publication by Allem et al. [23].

## Data Availability

Not applicable.

## References

[1] Guaman M.C., Pishevar N., Abman S.H., Keszler M., Truog W.E., Panitch H., Nelin L.D. (2021). Invasive mechanical ventilation at 36 weeks post-menstrual age, adverse outcomes with a comparison of recent definitions of bronchopulmonary dysplasia. J. Perinatol..

[2] Bancalari E., Jain D. (2019). Bronchopulmonary Dysplasia: 50 Years after the Original Description. Neonatology.

[3] Hernandez L.A., Peevy K.J., Moise A.A., Parker J.C. (1989). Chest wall restriction limits high airway pressure-induced lung injury in young rabbits. J. Appl. Physiol..

[4] Lavoie P.M., Pham C., Jang K.L. (2008). Heritability of bronchopulmonary dysplasia, defined according to the consensus statement of the national institutes of health. Pediatrics.

[5] Laughon M., Allred E.N., Bose C., O’Shea T.M., Van Marter L.J., Ehrenkranz R.A., Leviton A. (2009). Patterns of respiratory disease during the first 2 postnatal weeks in extremely premature infants. Pediatrics.

[6] Vento M., Moro M., Escrig R., Arruza L., Villar G., Izquierdo I., Roberts L.J., Arduini A., Escobar J.J., Asensi M.A. (2009). Preterm resuscitation with low O_2_ causes less oxidative stress, inflammation, and chronic lung disease. Pediatrics.

[7] Abman S.H., Bancalari E., Jobe A. (2017). The Evolution of Bronchopulmonary Dysplasia after 50 Years. Am. J. Respir. Crit. Care Med..

[8] Ryan R.M. (2006). A new look at bronchopulmonary dysplasia classification. Journal of perinatology. Off. J. Calif. Perinat. Assoc..

[9] Northway W.H., Rosan R.C., Porter D.Y. (1967). Pulmonary disease following respirator therapy of hyaline-membrane disease. Bronchopulmonary dysplasia. New Engl. J. Med..

[10] Bancalari E., Abdenour G.E., Feller R., Gannon J. (1979). Bronchopulmonary dysplasia: Clinical presentation. J. Pediatr..

[11] Shennan A.T., Dunn M.S., Ohlsson A., Lennox K., Hoskins E.M. (1988). Abnormal pulmonary outcomes in premature infants: Prediction from O_2_ requirement in the neonatal period. Pediatrics.

[12] Jobe A.H., Bancalari E. (2001). Bronchopulmonary dysplasia. Am. J. Respir. Crit. Care Med..

[13] Higgins R.D., Jobe A.H., Koso-Thomas M., Bancalari E., Viscardi R.M., Hartert T.V., Ryan R.M., Kallapur S.G., Steinhorn R.H., Konduri G.G. (2018). Bronchopulmonary Dysplasia: Executive Summary of a Workshop. J. Pediatr..

[14] Ehrenkranz R.A., Walsh M.C., Vohr B.R., Jobe A.H., Wright L.L., Fanaroff A.A., Wrage L.A., Poole K. (2005). Validation of the National Institutes of Health consensus definition of bronchopulmonary dysplasia. Pediatrics.

[15] Gien J., Baker C.D., Zhang H., Austin E.D., Collaco J.M., Guaman M.C. (2015). Point Prevalence, Clinical Characteristics, and Treatment Variation for Infants with Severe Bronchopulmonary Dysplasia. Am. J. Perinatol..

[16] Jensen E.A., Dysart K., Gantz M.G., McDonald S., Bamat N.A., Keszler M., Kirpalani H., Laughon M.M., Poindexter B.B., Duncan A.F. (2019). The Diagnosis of Bronchopulmonary Dysplasia in Very Preterm Infants. An Evidence-based Approach. Am. J. Respir. Crit. Care Med..

[17] Pierro M., Van Mechelen K., van Westering-Kroon E., Villamor-Martínez E., Villamor E. (2022). Endotypes of Prematurity and Phenotypes of Bronchopulmonary Dysplasia: Toward Personalized Neonatology. J. Pers. Med..

[18] El-Khuffash A., Mullaly R., McNamara P.J. (2023). Patent ductus arteriosus, bronchopulmonary dysplasia and pulmonary hypertension-a complex conundrum with many phenotypes?. Pediatr. Res..

[19] Wu K.Y., Jensen E.A., White A.M., Wang Y., Biko D.M., Nilan K., Fraga M.V., Mercer-Rosa L., Zhang H., Kirpalani H. (2020). Characterization of Disease Phenotype in Very Preterm Infants with Severe Bronchopulmonary Dysplasia. Am. J. Respir. Crit. Care Med..

[20] Tracy M.C., Cornfield D.N. (2020). Bronchopulmonary Dysplasia: Then, Now, and Next. Pediatr. Allergy Immunol. Pulmonol..

[21] Wang S.H., Tsao P.N. (2020). Phenotypes of Bronchopulmonary Dysplasia. Int. J. Mol. Sci..

[22] (2018). VON. Manual of Operations: Part 2. Data Definitions & Infant Data. Publisher Vermont Oxford Network. https://vtoxford.zendesk.com/hc/en-us/article_attachments/360024732954/Manual_of_Operations_Part_2_v23.2.pdf.

[23] Aleem S., Do M.B.T., Gantz M., Hibbs A.M., Jensen E.A., Cotten C.M., Malcolm W., Walsh M., Greenberg R.G. (2021). Assessing 3 Bronchopulmonary Dysplasia Definitions: Associations between Room Air Challenge Results and Respiratory Outcomes. Pediatrics.

[24] Dassios T., Curley A., Morley C., Ross-Russell R. (2015). Using Measurements of Shunt and Ventilation-to-Perfusion Ratio to Quantify the Severity of Bronchopulmonary Dysplasia. Neonatology.

[25] Cuevas Guaman M., Dahm P.H., Welty S.E. (2021). The challenge of accurately describing the epidemiology of bronchopulmonary dysplasia (BPD) based on the various current definitions of BPD. Pediatr. Pulmonol..

[26] Jensen E.A., Edwards E.M., Greenberg L.T., Soll R.F., Ehret D.E.Y., Horbar J.D. (2021). Severity of Bronchopulmonary Dysplasia Among Very Preterm Infants in the United States. Pediatrics.

[27] Schulzke S.M., Kaempfen S., Patole S.K. (2014). Pentoxifylline for the prevention of bronchopulmonary dysplasia in preterm infants. Cochrane Database Syst. Rev..

[28] Speer E.M., Dowling D.J., Xu J., Ozog L.S., Mathew J.A., Chander A., Yin D., Levy O. (2018). Pentoxifylline, dexamethasone and azithromycin demonstrate distinct age-dependent and synergistic inhibition of TLR- and inflammasome-mediated cytokine production in human newborn and adult blood in vitro. PLoS ONE.

[29] Jobe A.H. (2003). An unanticipated benefit of the treatment of preterm infants with CuZn superoxide dismutase. Pediatrics.

[30] Gentyala R.R., Ehret D., Suresh G., Soll R. (2019). Superoxide dismutase for preventing bronchopulmonary dysplasia (BPD) in preterm infants. Cochrane Database Syst. Rev..

[31] Bamat N.A., Kirpalani H., Feudtner C., Jensen E.A., Laughon M.M., Zhang H., Monk H.M., Passarella M., Lorch S.A. (2019). Medication use in infants with severe bronchopulmonary dysplasia admitted to United States children’s hospitals. J. Perinatol..

[32] Slaughter J.L., Stenger M.R., Reagan P.B. (2013). Variation in the use of diuretic therapy for infants with bronchopulmonary dysplasia. Pediatrics.

[33] Sindelar R., Shepherd E.G., Ågren J., Panitch H.B., Abman S.H., Nelin L.D. (2021). Bronchopulmonary Dysplasia Collaborative. Established severe BPD: Is there a way out? Change of ventilatory paradigms. Pediatr. Res..

[34] Katakam L., Suresh G.K. (2021). The diagnosis of bronchopulmonary dysplasia in very preterm infants-Which is the better definition?. Acta Paediatr..

[35] Walsh M., Engle W., Laptook A., Kazzi S.N.J., Buchter S., Rasmussen M., Yao M., For the National Institute of Child Health and Human Development Neonatal Research Network (2005). O_2_ delivery through nasal cannulae to preterm infants: Can practice be improved?. Pediatrics.

[36] Benaron D.A., Benitz W.E. (1994). Maximizing the stability of O_2_ delivered via nasal cannula. Arch. Pediatr. Adolesc. Med..

[37] Finer N.N., Bates R., Tomat P. (1996). Low flow O_2_ delivery via nasal cannula to neonates. Pediatr. Pulmonol..

[38] Jung Y.H., Jang J., Kim H.S., Shin S.H., Choi C.W., Kim E.K., Kim B.I. (2019). Respiratory severity score as a predictive factor for severe bronchopulmonary dysplasia or death in extremely preterm infants. BMC Pediatr..

[39] Malkar M.B., Gardner W.P., Mandy G.T., Stenger M.R., Nelin L.D., Shepherd E.G., Welty S.E. (2015). Respiratory severity score on day of life 30 is predictive of mortality and the length of mechanical ventilation in premature infants with protracted ventilation. Pediatr. Pulmonol..

[40] Madan A., Brozanski B.S., Cole C.H., Oden N.L., Cohen G., Phelps D.L. (2005). A pulmonary score for assessing the severity of neonatal chronic lung disease. Pediatrics.

[41] Committee on Fetus and Newborn (2002). Postnatal corticosteroids to treat or prevent chronic lung disease in preterm infants. Pediatrics.

[42] Cummings J.J., Pramanik A.K., Committee on Fetus and Newborn (2022). Postnatal Corticosteroids to Prevent or Treat Chronic Lung Disease following Preterm Birth. Pediatrics.

